# Supplementary material for: Resolving the full spectrum of human genome variation using Linked-Reads
Source: Genome Res. 2019 Apr;29(4):635–45. doi: 10.1101/gr.234443.118 (PMC6442396; doi:10.1101/gr.234443.118)
Supplement: Supplemental Material [file supp_gr.234443.118_Supplemental_longranger-2.2.zip › longranger-2.2.tar.bz2!/lr2.2-public/lib/stan/cmdstan/stan/doc/api/html/classstan_1_1mcmc_1_1base__adapter.html]

Stan: stan::mcmc::base\_adapter Class Reference


|  |  |
| --- | --- |
|  | Stan  2.5.0  probability, sampling & optimization |


- Main Page
- Related Pages
- Namespaces
- Classes
- Files

- Class List
- Class Index
- Class Hierarchy
- Class Members

All Classes Namespaces Files Functions Variables Typedefs Enumerations Enumerator Friends Macros Pages

- stan
- mcmc
- base\_adapter

Public Member Functions |
Protected Attributes |
List of all members

stan::mcmc::base\_adapter Class Reference

`#include <base_adapter.hpp>`

Inheritance diagram for stan::mcmc::base\_adapter:

|  |  |
| --- | --- |
| Public Member Functions | |
|  | base\_adapter () |
|  | |
| virtual void | engage\_adaptation () |
|  | |
| virtual void | disengage\_adaptation () |
|  | |
| bool | adapting () |
|  | |

|  |  |
| --- | --- |
| Protected Attributes | |
| bool | adapt\_flag\_ |
|  | |

## Detailed Description

Definition at line 8 of file base\_adapter.hpp.

## Constructor & Destructor Documentation

|  |  |  |  |  |
| --- | --- | --- | --- | --- |
| stan::mcmc::base\_adapter::base\_adapter | ( |  | ) |  |

inline

Definition at line 12 of file base\_adapter.hpp.

## Member Function Documentation

|  |  |  |  |  |
| --- | --- | --- | --- | --- |
| bool stan::mcmc::base\_adapter::adapting | ( |  | ) |  |

inline

Definition at line 17 of file base\_adapter.hpp.

|  |  |  |  |  |
| --- | --- | --- | --- | --- |
| virtual void stan::mcmc::base\_adapter::disengage\_adaptation | ( |  | ) |  |

inlinevirtual

Reimplemented in stan::mcmc::adapt\_dense\_e\_static\_hmc< M, BaseRNG >, stan::mcmc::adapt\_diag\_e\_static\_hmc< M, BaseRNG >, stan::mcmc::adapt\_dense\_e\_nuts< M, BaseRNG >, stan::mcmc::adapt\_diag\_e\_nuts< M, BaseRNG >, stan::mcmc::adapt\_unit\_e\_static\_hmc< M, BaseRNG >, and stan::mcmc::adapt\_unit\_e\_nuts< M, BaseRNG >.

Definition at line 15 of file base\_adapter.hpp.

|  |  |  |  |  |
| --- | --- | --- | --- | --- |
| virtual void stan::mcmc::base\_adapter::engage\_adaptation | ( |  | ) |  |

inlinevirtual

Definition at line 14 of file base\_adapter.hpp.

## Member Data Documentation

bool stan::mcmc::base\_adapter::adapt\_flag\_

protected

Definition at line 21 of file base\_adapter.hpp.

---

The documentation for this class was generated from the following file:

- src/stan/mcmc/base\_adapter.hpp


---

[ Stan Home Page ]

*© 2011–2014,
Stan Development Team.*
